# Supplementary material for: Investigating the Potential of Division of Labor in Synthetic Bacterial Communities for the Production of Violacein
Source: ACS Synth Biol. 2025 Jun 17;14(7):2703–9. doi: 10.1021/acssynbio.5c00120 (PMC12281613; doi:10.1021/acssynbio.5c00120)
Supplement: Supplementary file 1 [file sb5c00120_si_001.pdf]

Investigating the Potential of Division of Labour in Synthetic Bacterial Communities for the Production of Violacein

Harman Mehta[1], Jose Jimenez[2], Rodrigo Ledesma-Amaro[1,3,4*], and Guy-Bart Stan[1*]

1 Department of Bioengineering, Imperial College London, London SW7 2AZ, UK
2 Department of Life Sciences, Imperial College London, London SW7 2AZ, UK
3 Bezos Centre for Sustainable Protein, Imperial College London, London SW7 2AZ, UK
4 UKRI Engineering Biology Mission Hub on Microbial Food, Imperial College London, London SW7 2AZ, UK
*Corresponding authors: Guy-Bart Stan(g.stan@imperial.ac.uk), Rodrigo Ledesma-Amaro(r.ledesma-amaro@imperial.ac.uk)

SupplementaryInformation

Supplementary Table 1 Plasmids used in this study

Plasmid No	Plasmid Name	Description	Antibiotic
pHM068	TrpED_sfGFP_pAB351	TetRpTet-RBSc33-TrpED-T1+J23116-RBSc33-sfGFP-T1 in pAB351	Amp
pHM053	VioABEDC_pStA212	T7(G6)-VioA-T7(G6)-VioB-T7(G6)-VioE-T7(G6)-VioC-T7(G6)-VioD in pStA212	Km
pHM063	VioABE_pStA212	T7(G6)-VioA-T7(G6)-VioB-T7(G6)-VioE in pStA212	Km
pHM066	VioDC_pStA212	T7(G6)-VioC-T7(G6)-VioD in pStA212	Km
pHM064	VioABED_pStA212	T7(G6)-VioA-T7(G6)-VioB-T7(G6)-VioE-T7(G6)-VioD in pStA212	Km
pHM065	VioC_pStA212	T7(G6)-VioC in pStA212	Km
pHM120	mRFP_pJBL5945	J23116-RBSc33-mRFP-T1 in pJBL5945	Amp

Supplementary Table 2 Strains used in this study

Strain	Description	Reference	Source
DH10B	F⁻ *mcrA* Δ(*mrr-hsdRMS-mcrBC*) φ80 *lacZ* ΔM15 Δ*lacX74 recA1 endA1 araD139* Δ(*ara-leu*)7697 *galU galK* λ⁻ *rpsL* (Str^r) *nupG*		
DH5α	F⁻ φ80 *lacZΔM15* Δ(*lacZYA-argF*) *U169 recA1 endA1 hsdR17* (rK⁻, mK⁺) *phoA supE44* λ⁻ *thi-1 gyrA96 relA1*		
BL21(DE3)	F⁻ *ompT hsdSB* (rB⁻ mB⁻) *gal dcm* (DE3)		
B6	F⁻ *ompT hsdSB* (rB⁻ mB⁻) *gal dcm* (DE3) Δ*trpR* Δ*tnaA*		(Fang et al., 2015)
Trp	pHM068 pStA212 TrpED_pTet_TetR + sfGFP pAB351 in B6	HM_174	This study
VioABE	pHM063 pHM068 VioABE TrpED in B6	HM_139	This study
VioABED	pHM064 pHM068 VioABED TrpED in B6	HM_142	This study
VioABEDC (Monoculture)	pHM053 pHM068 VioABECD TrpED in B6	HM_145	This study
VioC	pHM065 pHM120 VioC mRFP in BL21(DE3)	HM_262	This study
VioD	pHM066 pHM120 VioCD mRFP in BL21(DE3)	HM_263	This study
VioABEDC	pHM053 pHM120 VioABECD mRFP in BL21(DE3)	HM_264	This study

Supplementary Table 3 Oligos used in this study

Oligo No	Oligo Name	Sequence
HM_030	TrpED_Fwd	AAGGGGTTGGTCTCATGTGGCTCTTCGATGATGCAAAC ACAAAAACCGACTCTCG
HM_031	TrpED_Rev	CAGTGTTGGGTCTCTGGTCGCTCTTCATTACCCTCGTG CCGCCAGTG
HM_032	Ser40LeuT_ Fwd	GCTGCTGGAATTCGCAGATATCGAC
HM_033	Ser40LeuT_ Rev	GTCGATATCTGCGAATTCCAGCAGC
HM_034	Met293Thr C_Fwd	CCCAGCCCGTACACGTTTTTATGCAG
HM_035	Met293Thr C_Rev	CTGCATAAAAACGTGTACGGGCTGGG
HM_065	212FA	GAATGGTCTCAGGAGCACGGCGTAGCCTAGGATC
HM_066	212RD	CAAATGGTCTCTAGTATCCATAGTCGACTCCTCCTTTC GC
HM_067	ABFA	AAGGGGTTGGTCTCATGTGGCTCTTCGCAGGTCCGGCG TAGCCTAGGATCGAG
HM_068	BZFD	AAGGGGTTGGTCTCATGTGGCTCTTCGCAGGGGAGAAT GAATTAACTCGAGTC
HM_069	ABFC	AAGGGGTTGGTCTCATGTGGCTCTTCGCAGCTCTCATA GGAATTGACTCGAG
HM_070	212FC	GAATGGTCTCAGGAGCAGCTCTCATAGGAATTGACTC GAG
HM_001	VioA_Fwd	AAGGGGTTGGTCTCATGTGGCTCTTCGATGTTAAATTT AGAGCATTCAGTTTC
HM_002	VioA_Rev	CAGTGTTGGGTCTCTGGTCGCTCTTCATTATCAAAGGT ATACTACTTCTTTCACAG
HM_003	VioB_Fwd	AAGGGGTTGGTCTCATGTGGCTCTTCGATGAGTGTTTT AGATTTTCCTCG
HM_004	VioB_Rev	CAGTGTTGGGTCTCTGGTCGCTCTTCATTACTAACCTT CCTTTGAAAGAGTTC
HM_005	VioC_Fwd	AAGGGGTTGGTCTCATGTGGCTCTTCGATGAGTAAAAT AATTATTGTTGGTGG
HM_006	VioC_Rev	CAGTGTTGGGTCTCTGGTCGCTCTTCATTAATTCATTCT CCCTATTTTGTACC
HM_007	VioD_Fwd	AAGGGGTTGGTCTCATGTGGCTCTTCGATGAACATTTT AGTGATCGGGGC
HM_008	VioD_Rev	CAGTGTTGGGTCTCTGGTCGCTCTTCATTAACGTTGCA GCGCATAACC
HM_009	VioE_Fwd	AAGGGGTTGGTCTCATGTGGCTCTTCGATGGAATTACG TAAAGTAGATAGAG
HM_010	VioE_Rev	CAGTGTTGGGTCTCTGGTCGCTCTTCATTATCAATTCCT ATGAGAGAGACTTG

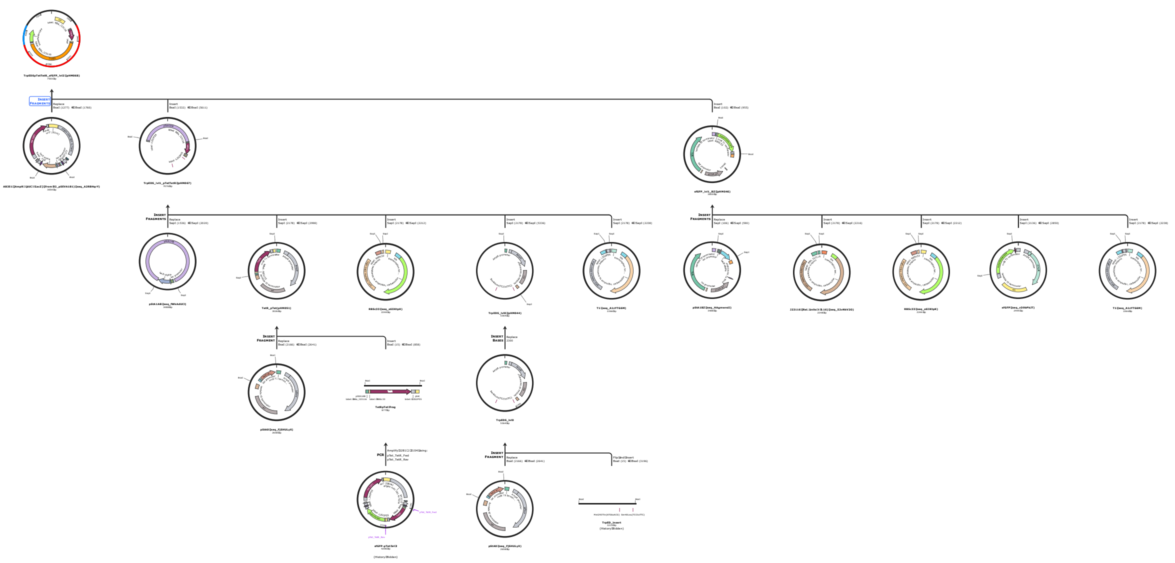

Supplementary Figure 1. Cloning strategy for constructing plasmid TrpED_sfGFP_pAB351 (pHM068).

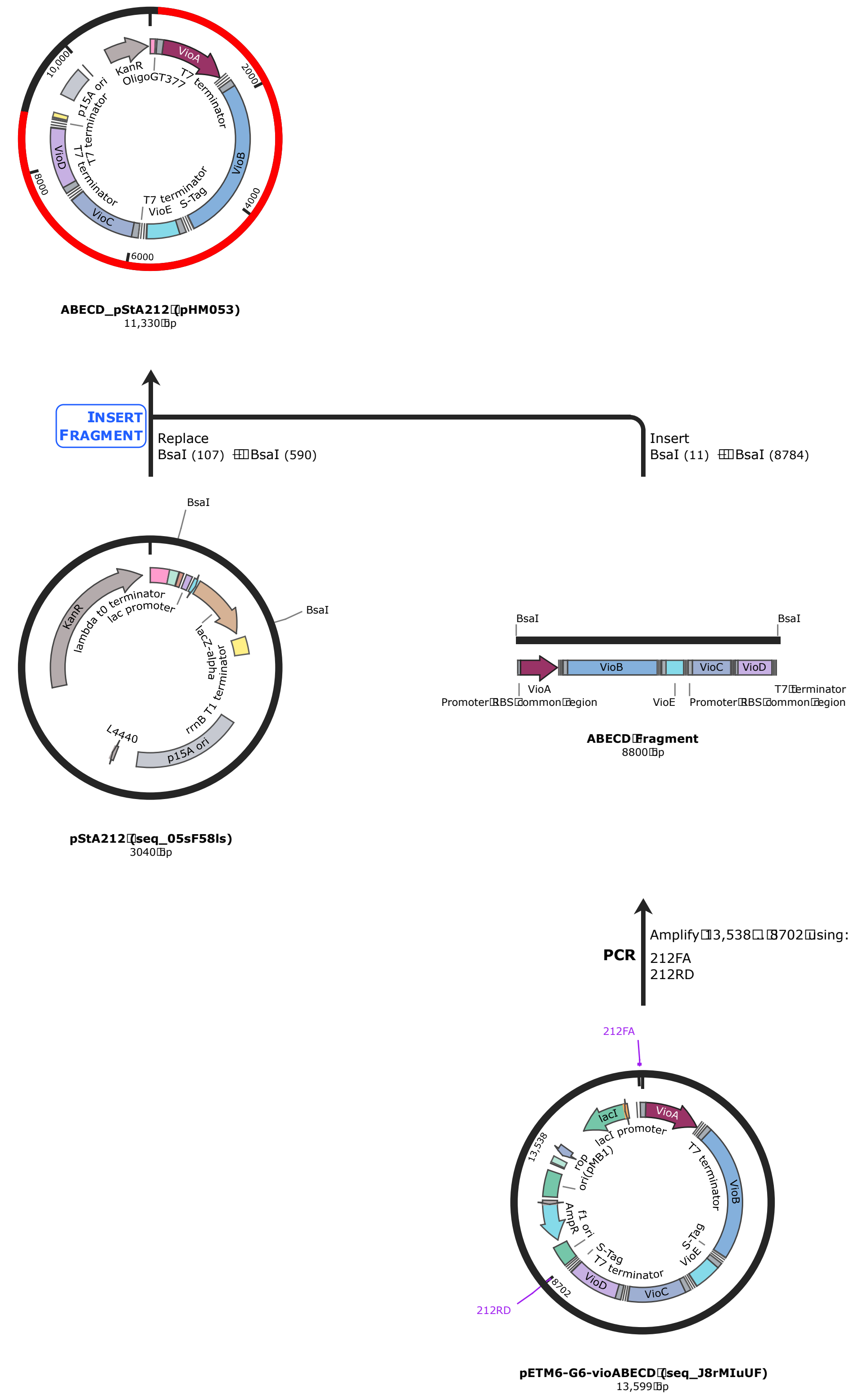

Supplementary Figure 2. Cloning strategy for constructing plasmid VioABEDC_pStA212 (pHM053)

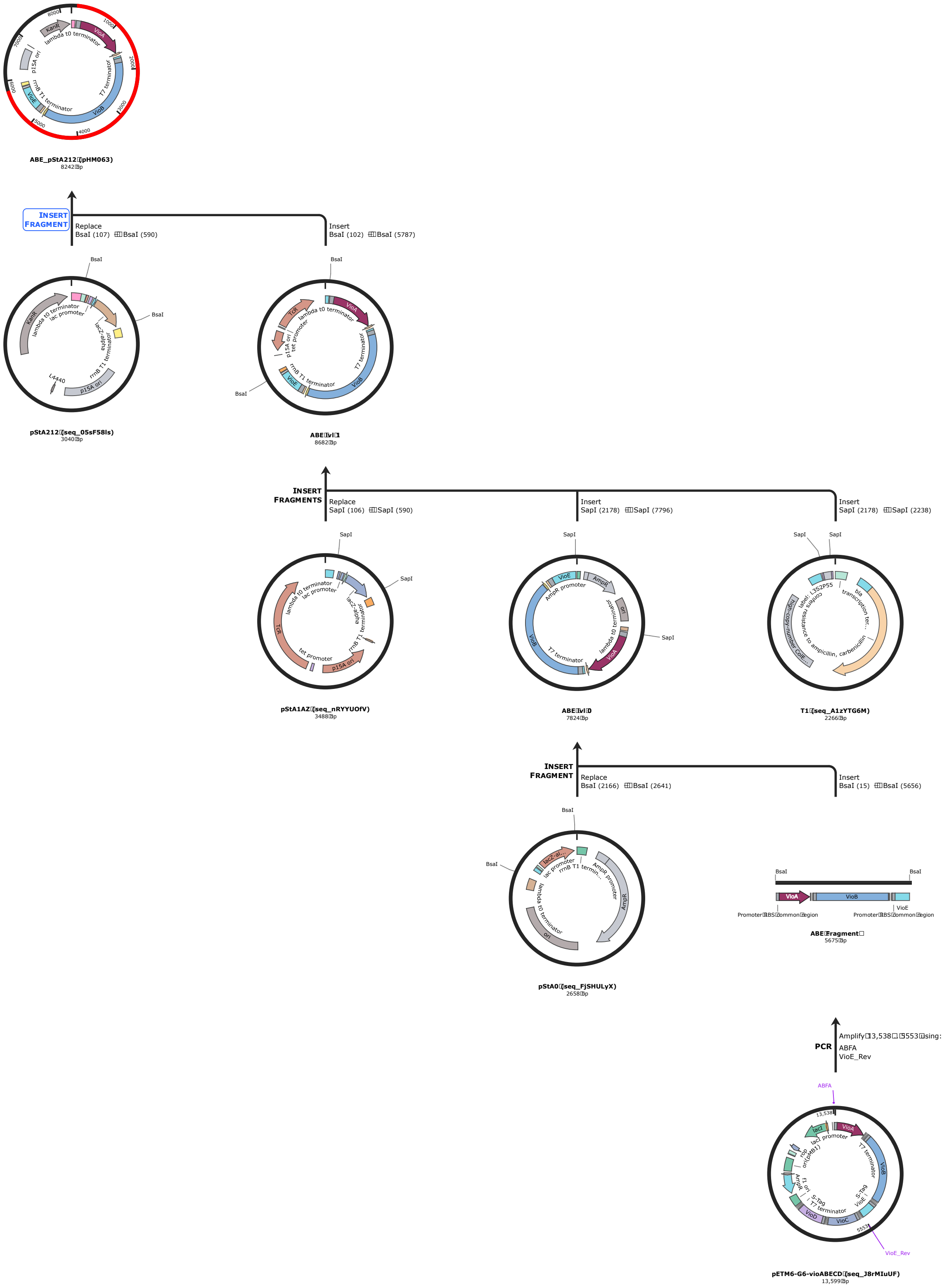

Supplementary Figure 3. Cloning strategy for constructing plasmid VioABE_pStA212 (pHM063)

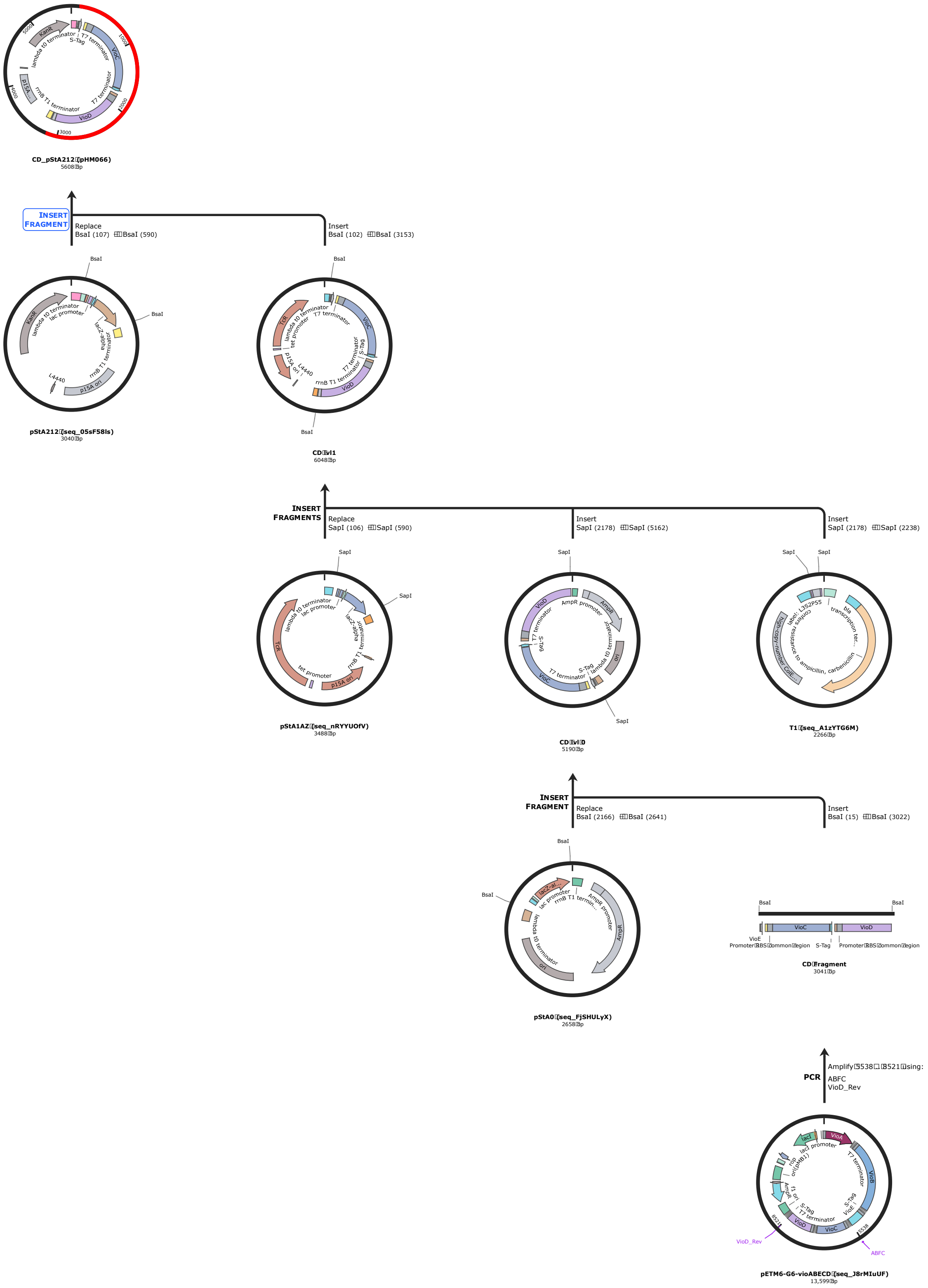

Supplementary Figure 4. Cloning strategy for constructing plasmid VioDC_pStA212 (pHM066)

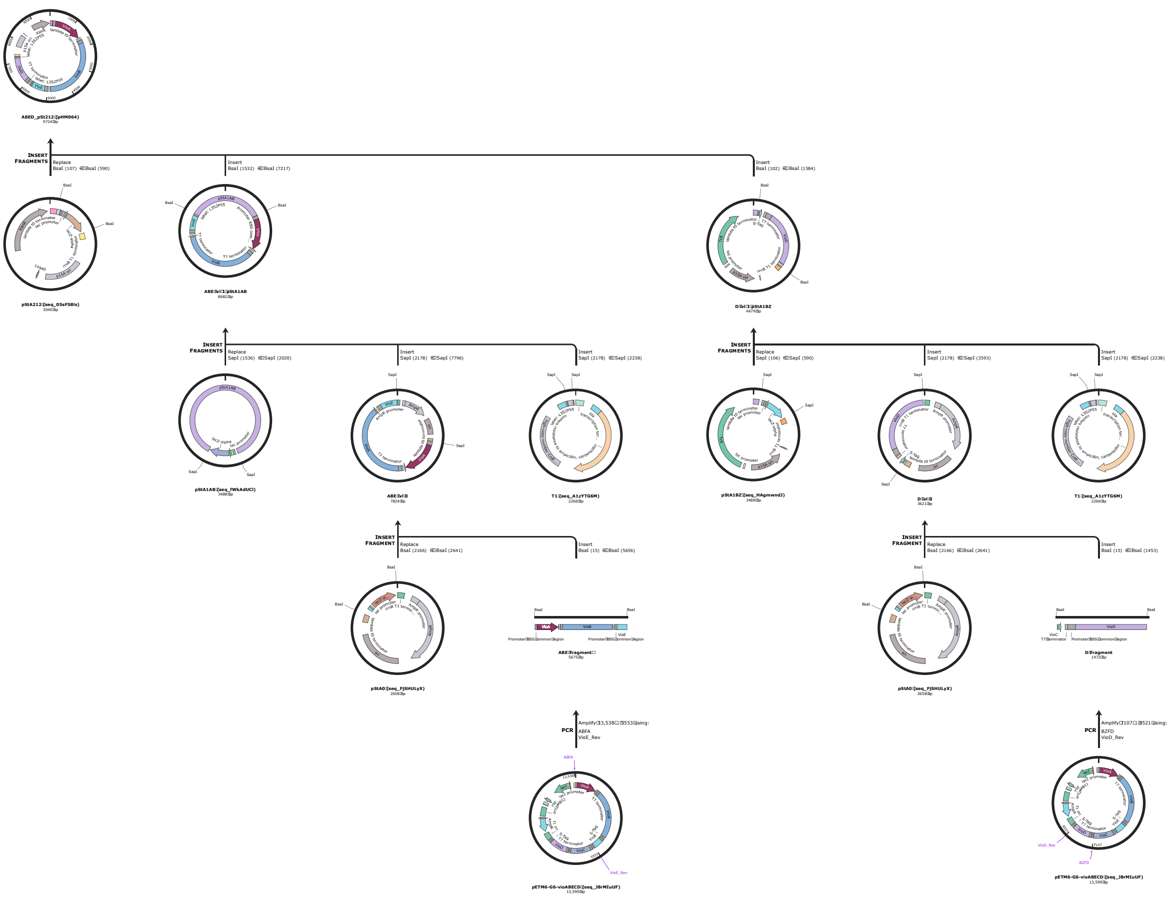

Supplementary Figure 5. Cloning strategy for constructing plasmid VioABED_pStA212 (pHM064)

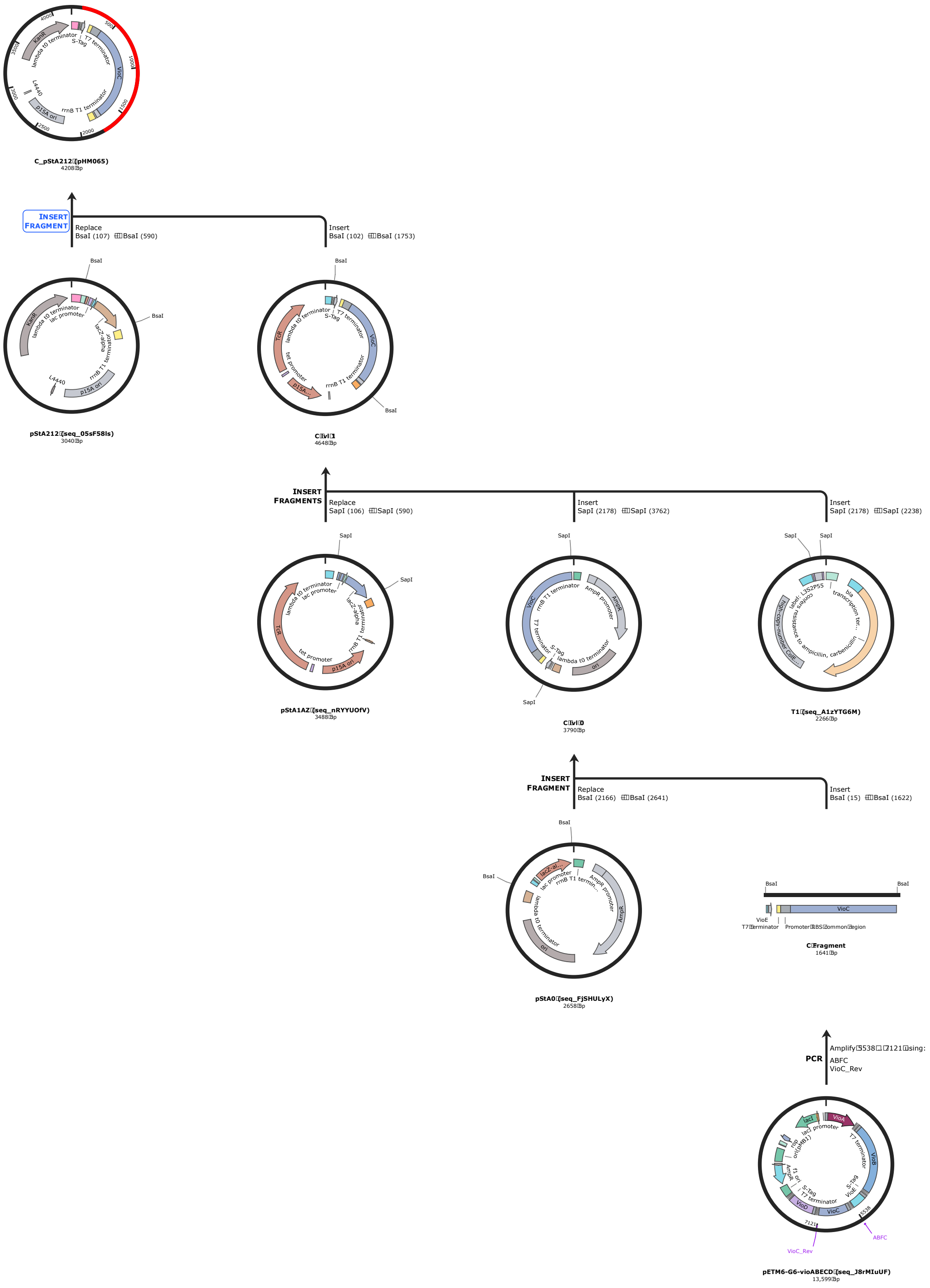

Supplementary Figure 6. Cloning strategy for constructing plasmid VioC_pStA212 (pHM065)

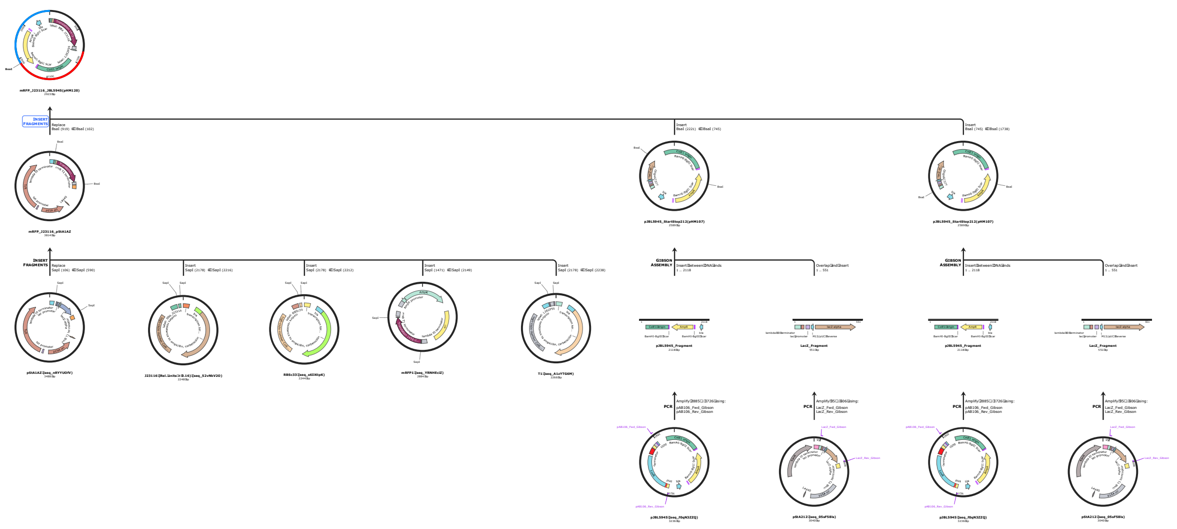

Supplementary Figure 7. Cloning strategy for constructing plasmid mRFP_pJBL5945 (pHM120)

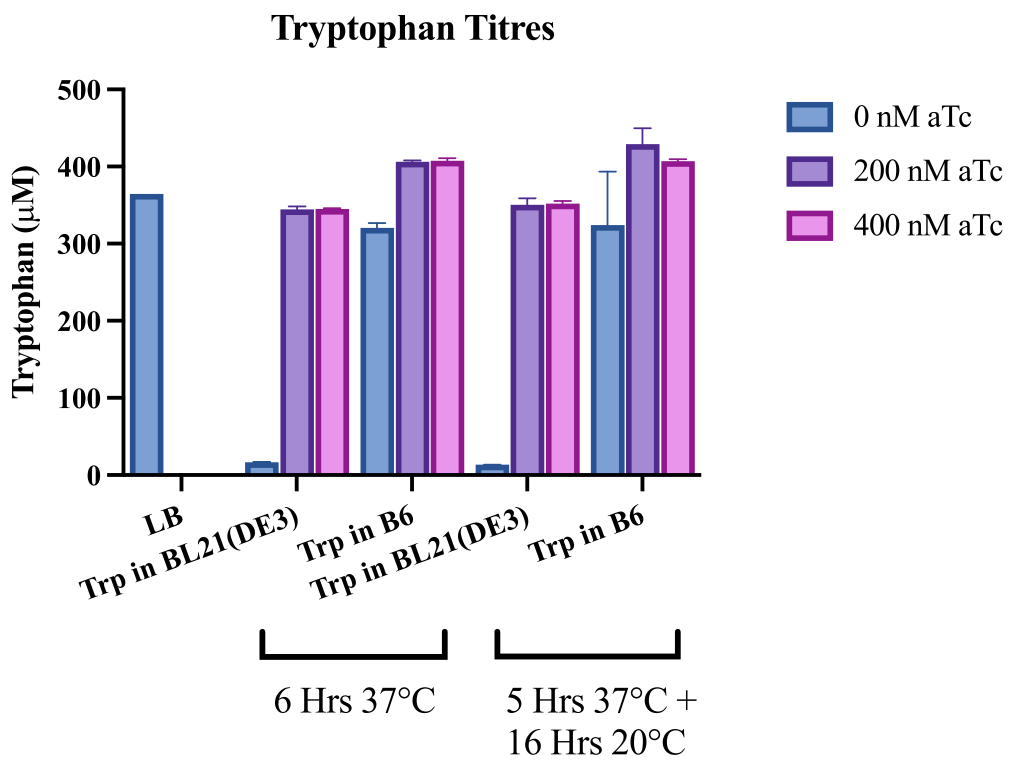

Supplementary Figure 8. Tryptophan production by the tryptophan overproducing strain developed. The tryptophan overproducing plasmid, pHM068, was transformed in both wild type BL21(DE3) strain and the tryptophan accumulating mutant strain B6. 2 mL of liquid cultures were incubated in 14mL culture tubes under two conditions, 6 hours at 37°C and 5 hours at 37°C followed by 16 hours at 20°C. Following this, the cells were disrupted and the supernatant was collected for tryptophan measurement by HPLC. Values shown are n=2 biological replicates with mean value shown and error bars representing standard deviation.

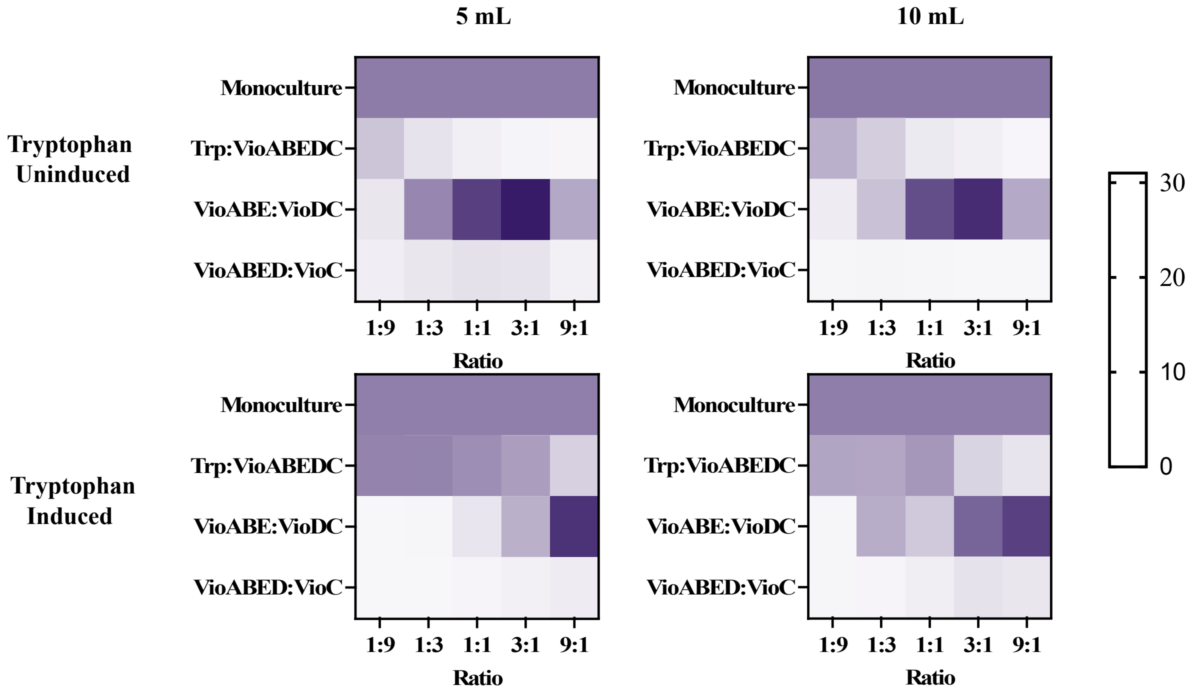

Supplementary Figure 9. Violacein titres produced by cocultures with different pathway split and different initial composition. The cocultures were tested at different culture volumes, 5 mL of liquid culture in 14 mL culture tubes and 10 mL of liquid culture in 50 mL shake flasks. The experiment was also repeated with and without the induction of the tryptophan overproduction genes. Values shown are mean violacein titres of three biological replicates.

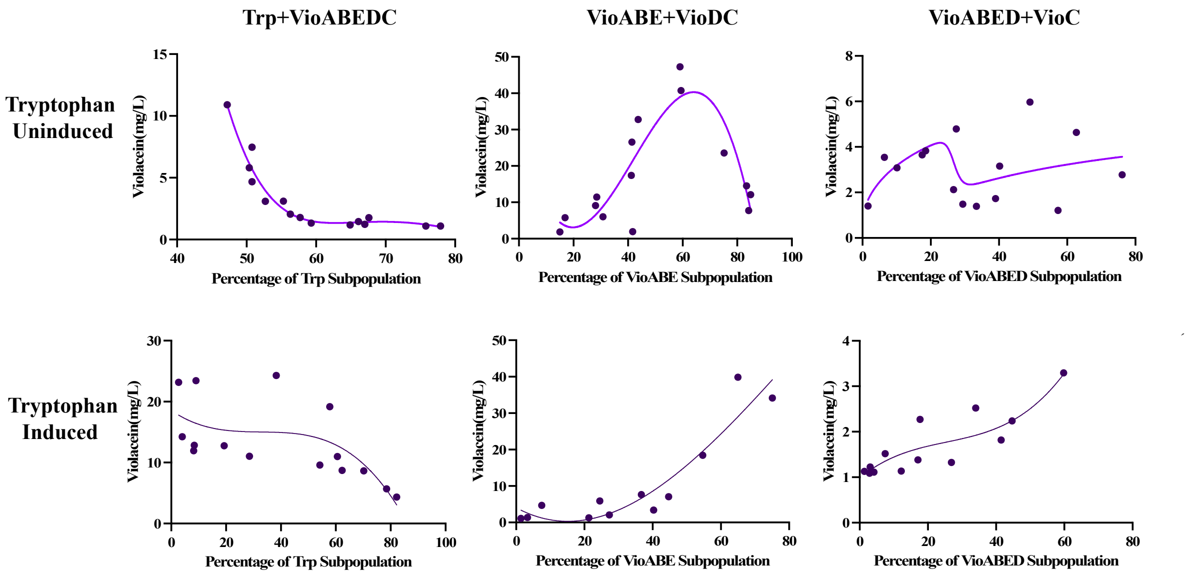

Supplementary Figure 10. Relationship between violacein tires and final coculture compositions for the different coculture splits, with and without the induction of the tryptophan overproducing genes. Lines represent nonlinear (third order polynomial) fit for the data. The R squared values for the curves in the six plots respectively are: 0.95, 0.74, ,0.46, 0.88 and 0.77.

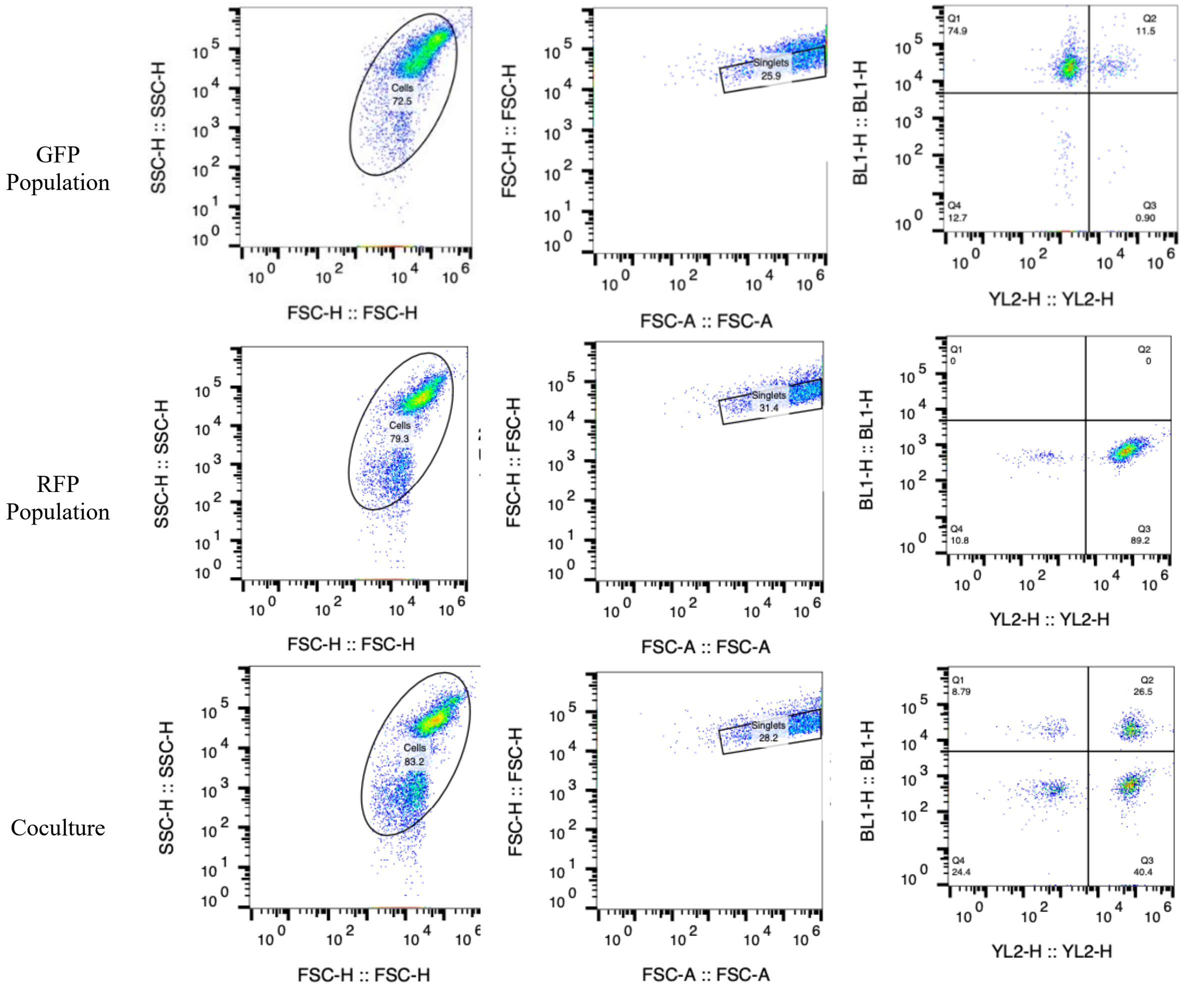

Supplementary Figure 11. Gating strategy applied for determining the coculture composition. The BL1 channel identifies the GFP tagged population while the YL2 channel identifies the RFP tagged population. The population showing positive for both RFP and GFP was identified to be cells sticking together and was hence the Q2 events were distributed equally to the GFP and RFP populations. Three samples shown here are for a sfGFP tagged population, mRFP tagged population and a coculture composed of the two subpopulations.
